# Comparing Operationalized Approaches for Substantial Reduction of Functioning in Chronic Fatigue Syndrome and Myalgic Encephalomyelitis

**DOI:** 10.36959/547/653

**Published:** 2022-04-21

**Authors:** Elzbieta Wiedbusch, Leonard A Jason

**Affiliations:** 1 DePaul University, USA

**Keywords:** Fatigue assessment, Operationalization, Diagnostic approach, Comparative analysis, Chronic Fatigue Syndrome, Myalgic Encephalomyelitis

## Abstract

A core criterion for Chronic Fatigue Syndrome (CFS) and Myalgic Encephalomyelitis (ME) is a substantial reduction in functioning from pre-illness levels. Despite its ubiquity in diagnostic criteria, there is considerable debate regarding how to measure this domain. The current study assesses five distinct methods for measuring substantial reductions. The analysis used an international, aggregated dataset of patients (N = 2,368) and controls (N=359) to compare the effectiveness of each method. Four methods involved sophisticated analytic approaches using the Medical Outcomes Survey Short Form-36; the fifth method included a single self-report item on the DePaul Symptom Questionnaire (DSQ). Our main finding was that all methods produced comparable results, though the DSQ item was the most valid in differentiating patients from controls. Having a simple, reliable method to capture a substantial reduction in functioning has considerable advantages for patients and health care workers.

## Introduction

Most case definitions for Chronic Fatigue Syndrome (CFS) and Myalgic Encephalomyelitis (ME) include a substantial reduction in functioning from pre-illness activity levels. Since many individuals experience fatigue resulting in reduced functioning at least once in their lifetime, it is critical to have an operationalized definition of substantial reduction that differentiates patients from healthy controls with fatigue [1].

Unfortunately, there is little guidance or consensus on how to measure these reductions or this construct. For example, the Fukuda, et al. (1994) [2] CFS criteria require substantial reductions in previous functioning levels but do not provide information on how to determine previous or current functioning in patients. More recently, the Institute of Medicine (IOM, 2015) constructed a new clinical case definition that requires a significant decrease in pre-illness levels of occupational, educational, social, or personal activities. Although the IOM (2015) did include more scientific and measurable guidelines, there is no consensus on which measures and assessment scores indicate acute reductions in functioning. Consequently, this criterion has varied across researchers and medical professionals.

There have been efforts made to operationalize the substantial reduction criteria, such as Reeves, et al. (2005) [3], who developed an empiric CFS case definition. They defined a substantial reduction in functioning using the Medical Outcomes Study 36-Item Short-Form Health Questionnaire (MOS SF-36) as a score at or below the 25th percentile of the general population, on at least one of the following subscales: Physical Functioning (≤ 70), Role-Physical (≤ 50), Social Functioning (≤ 75), and Role-Emotional (≤ 66.7) [3]. Jason, et al. (2009) later found that individuals with clinical depression would meet the disability criterion for CFS because of the Role-Emotional subscale and criticized this definition. This empiric definition could misclassify individuals with Major Depressive Disorder as patients with CFS. Furthermore, it remains unclear how those four subscales were chosen and why only one of the four subscales could establish a substantial reduction in patients.

In response to these issues, Jason, et al. (2011) [4] utilized a Receiver Operating Characteristic (ROC) curve analysis to evaluate the MOS SF-36 subscales and determine optimal cut-off scores. They found that the subscales with the best sensitivity and specificity for both tertiary and community-based ME/CFS samples were the Vitality, Social Functioning, and Role-Physical subscales. The study suggested the following optimal cut-off scores for substantial reduction: ≤ 35 Vitality, ≤ 62.5 Social Functioning, and ≤ 50 Role-Physical. Jason, et al. (2011) [4] also found that meeting at least two of the three cut-off scores for the respective subscales result in the best sensitivity and specificity for discriminating between ill and non-ill groups.

To assess the MOS SF-36 subscales and optimal cut-off scores for measuring a substantial reduction within a younger sample of patients with ME/CFS, Gleason, et al. (2018) [5] utilized the Youden Index (YI) (sensitivity + specificity-1) to calculate the optimal cut-off scores using the sensitivity and specificity levels. Among the young adult sample, the following cut-off scores maximized sensitivity and specificity on the scales examined: Physical Functioning ≤ 80 (YI=0.905), General Health ≤ 47 (YI = 0.828), Role Physical ≤ 25 (YI = 0.810), and Social Functioning ≤ 50 (YI = 0.764). Their analysis also found that meeting at least three out of the four cut-off scores in the respective subscales offered discrimination between ME/CFS and non-ill groups.

Although other physical illnesses do not require patients to prove impairments in previous levels of functioning, because it is in the ME/CFS case definitions, there is a need for consensus on how to measure a substantial reduction in functioning in patients with ME/CFS. There is evidence of the usefulness of this construct, as Becker et al. (2001) [6] found maximal workload at exhaustion averaged 53% of normal in their patients with ME/CFS, which is close to the 50% decrease in physical capabilities described in several criteria for ME/CFS. However, this type of testing is expensive and not all patients have access to tests to assess maximal workload. The current study explores different methods for measuring a substantial reduction within patients with ME/CFS. The first four out of five methods use the subscales in the SF-36 and the cut-off scores from the analyses done by Jason, et al. (2011) [4] and Gleason, et al. (2018) [5]. The fifth method uses a single item from the DePaul Symptom Questionnaire (DSQ) to assess how well it differentiated patients from controls.

## Methods

### Participants

We employed an aggregated sample of patients with ME/CFS (n = 2,368) and controls (N = 359). Each of the data sets has been described [7–10] but below we provide brief descriptions.

#### DePaul Sample -

An international convenience sample of individuals who were at least 18 years old, with a self-reported, current diagnosis of ME/CFS. 216 individuals completed the assessment, and they were of average age of 52.0 (SD=11.3) years, 84.2% were female, 97.7% were White, 13% were working full- or part-time, 57.2% were on disability. 40.2% held a graduate or professional degree, 34.6% held a standard college degree, 18.2% had attended college for at least one year, and 7.0% completed high school or had a GED.

A control sample included 96 DePaul University students who were at least 18 years old. 70.8% were female, their average age was 20.6 years (SD=2.6), 60% were White, 13.7% were Asian or Pacific Islander, 9.5% were Black, and 15.8% indicated another race. 10.4% were working part-time.

#### Solve ME/CFS BioBank (2016) -

The SolveCFS Biobank involved a sample of individuals diagnosed by a physician specializing in ME/CFS. 505 patients were recruited, and the sample’s average age was 54.9 years (SD=12.1). They were 97.7% White and 77.0% female; 25.8% had at least one year of college, and 70.1% held a standard college degree. 20.6% were working full- or part-time, with 46.4% on disability. 53 participants were controls, of average age of 56.1 years (SD=13.0). 100% of the controls were White and 66.0% were female (66.0%). 27.5% were retired, and 66.7% worked either full- or part-time. 88.7% had a standard college degree.

#### Newcastle -

An experienced physician diagnosed participants with ME/CFS. The average age of the 100 patients was 45.8 years (SD=13.9), and 99.0% of the sample was White, 81.0% were female, 36.7% were working either full- or part-time, and 30.6% were on disability. 20.2% had a graduate or professional degree, 28.7% had a college degree, 24.5% had completed at least one year of college, and 13.8% had a high school degree or GED.

#### Norway 1 sample:

176 patients from southern Norway with a diagnosis of ME/CFS by a physician were recruited. 99.4% of the sample was White, 86.3% were female, with a mean age of 43.6 years (SD=11.9). 83.5% were on disability; 9.8% had a graduate or professional degree, 40.2% had a standard college degree, and 42.0% completed high school or had a GED.

#### Norway 2 sample:

Participants were from either an inpatient medical ward for severely ill patients or an outpatient clinic at a multidisciplinary ME/CFS center. Among the 64 participants, 96.8% were White, 81.3% were female, and their average age was 35.3 years (SD=11.9). 45.3% of the sample had completed high school or a GED, 25.0% had a standard college degree, and 12.5% had a graduate or professional degree. 82.8% were on disability.

#### Norway 3 sample:

A specialist at a ME/CFS tertiary care center recruited participants who meet the Canadian Consensus criteria for ME/CFS [11] from Oslo University Hospital. The sample of 175 patients were 97.1% White, 80.6% female, of a mean age of 38.4 years (SD=11.3). 33.5% of the sample completed high school or a GED, 40.5% held a standard college degree, and 15.6% held a graduate or professional degree. 90.0% were on disability.

First time blood donors referred by physicians at Oslo University Hospital represented the healthy control group in the Norway 3 sample. Participants were between 18–65 years old and fluent in the Norwegian language. Of the 210 participants, 96.2% were White, 68.6% were female, and their average age was 31.4 years (SD=8.5), 42.5% held a standard college degree, 28.9% held a graduate degree, and 28.5% completed high school or had a GED.

#### Chronic Illness sample:

Respondents were from a convenience sample of adults living with ME/CFS This sample included 407 participants who were 97.0% White, 88.2% female and of a mean age of 48.7 years (SD=13.1). 29.9% had a standard college degree and 40.2% had a graduate degree. 48.9% were on disability.

#### Japan sample:

This sample was recruited from the ME Japan Association and affiliated physician clinics specializing in ME/CFS. Of the 129 participants, 78.9% were female, and they were of a mean age of 46.2 years (SD=13.3). 41.7% had a standard college degree, 8.7% had a graduate degree, 17.3% completed at least one year of college, and 22.8% completed high school or had a GED. 26.3% of participants were working full- or part-time. 28.0% of the sample was on disability.

#### Spain sample:

232 participants meeting the 1994 Fukuda, et al. (1994) [2] case definition were recruited from a tertiary referral center in Barcelona, Spain. 98.9% were White, 85.7% were female, with a mean age of 50.4 years (SD=8.6). 40.0% had not completed high school, 25.9% had a high school diploma or GED, 18.9% completed at least a year of college, and 15.1% had at least a standard college degree.

#### Amsterdam sample:

364 patients were recruited from the outpatients clinic at the CFS Medical Center in Amsterdam. 77.9% were female with a mean age of 37.2 years (SD=11.5), 30.5% had a high school diploma or GED, 22.1% held a standard college degree, and 19.9% held a graduate or professional degree.

## Measures

### DePaul symptom questionnaire (DSQ)

The DSQ is a self-report measure of demographics; ME/CFS symptomatology; and medical, occupational, and social history [12]. This measure was developed to classify individuals by a variety of ME/CFS case definitions, but the 54 symptoms were based upon the Clinical Canadian ME/CFS criteria [11]. The DSQ has evidenced good test-retest reliability among both patient and control groups [13]. A factor analysis of these symptoms resulted in a three-factor solution, and these factors evidenced good internal consistency [14]. The DSQ is available in the shared library of Research Electronic Data Capture (REDCap) [15], hosted at DePaul University: https://redcap.is.depaul.edu/surveys/?s=tRxytSPVVw.

### Medical outcomes study 36-item short form health questionnaire (SF-36)

The SF-36 assesses physical and mental functioning [16], and is comprised of eight subscales: Physical Functioning, Role Physical, Bodily Pain, General Health, Social Functioning, Mental Health, Role Emotional, and Vitality. Higher scores indicate less disability. This instrument has strong psychometric properties, such as good internal consistency and discriminant validity [16, 17].

### Five methods to measure substantial reductions

There are five methods employed in the current study to operationalize substantial reduction. Four of these methods use the subscales found in the SF-36, while the fifth method utilizes one self-report item on the DSQ.

#### Method 1 -

Based on the suggestions made by Jason et al. (2011) [4], participants needed to meet two out of three of the following cut-offs to meet substantial reduction: Vitality ≤ 35, Social Functioning ≤ 62.5, and Role-Physical ≤ 50.

#### Method 2 -

Based on the study by Gleason and colleagues (2018) [5], participants needed to meet three out of four of the following cut-offs to meet substantial reduction: Physical Functioning ≤ 80, General Health ≤ 47, Role Physical ≤ 25, and Social Functioning ≤ 50.

#### Method 3 -

Based on the study by Gleason and colleagues (2018) [5], participants needed to meet at least two out of three scores to demonstrate substantial reduction: Physical Functioning ≤ 80, General Health ≤ 50, and Role-Physical ≤ 50 (a score of 50 or less of Role-Physical functioning was the best discriminator in community-based samples [4]).

#### Method 4 -

The fourth method, also recommended by Gleason and colleagues (2018) [5], required participants to meet at least three out of the four following cut-off scores to determine substantial reduction in functionality: Physical Functioning ≤ 80, General Health ≤ 50, Role Physical ≤ 50, and Social Functioning ≤ 62.5.

#### Method 5 -

The fifth method involved operationalizing substantial reduction from a single DSQ self-report item that asks, “Since the onset of your problems with fatigue/energy, have your symptoms caused a 50% or greater reduction in your activity level?” Individuals select “yes,” “no,” or “I do not have issues with fatigue/energy.” Physicians are often uncertain of a patient’s activity level prior to their onset of symptoms; this item allows patients to indicate whether they experienced an acute reduction in functioning based on their knowledge of prior and current activity levels.

## Results

As indicated in Table 1, Method 1 identified substantial reduction in 91.2% of patients with ME/CFS and 13.6% of controls. Method 2 captured the fewest patient*s*, with 89.0% of the patient sample and 4.7% of the controls. In Method 3, 92.2% of patients and 10.9% of controls met substantial reduction. Comparably, Method 4 identified 90.8% of the ME/CFS sample and 7.5% of controls. Finally, Method 5 identified 91.7% of patients and 6.4% of controls.

## Discussion

The current study assessed different criteria that measure substantial reductions in functioning. Our main finding was that all methods produced comparable results, suggesting that the method with the least burden to patients involves one-item from the DSQ. Adopting this method would simplify the procedure for assessing this domain contained within the major ME/CFS case definitions.

The DSQ’s item may allow researchers to avoid some of the current problems with case definitions and their varying guidelines. For example, in the Myalgic Encephalomyelitis International Consensus Case Criteria (ME-ICC), Carruthers and colleagues (2011) [18] operationalized substantial reduction into three severity categories. The “mild” level indicated an approximate 50% reduction in a patient; “moderate” suggested patients are mostly housebound; “severe” to “very severe” signified that a patient is mostly or entirely bedridden. The following year, Carruthers, et al. (2012) [19] developed a primer using the ME-ICC criteria for medical practitioners to diagnose patients with ME/CFS. However, the primer differed from the previous year’s guidelines in the ME-ICC [20]. The original description of this criteria in the ME-ICC [18] designates “mild” as an approximate 50% reduction, while the ME primer [19] defines the “moderate” level as a 50% reduction. It is evident that determining consistent standards for this criterion is challenging, even when case definitions attempt to operationalize these criteria.

It is worth noting that the Diagnostic and Statistical Manual of Mental Disorders primarily uses a substantial reduction criterion for diagnosing mental disorders [20]. Physical illnesses do not require patients to prove impairments in levels of functioning, so it is unclear whether this is necessary for those with ME/CFS. Indeed, the substantial reduction criterion may be stigmatizing to individuals with ME/CFS, as it denotes that patients must prove impairment to medical professionals [1, 9]. However, as it is part of almost all ME/CFS case definitions, finding appropriate ways to measure it is of importance for health care workers.

There are some limitations in the implications and methods of this study. Many patients with ME/CFS have neurocognitive difficulties, which could affect their ability to recall prior levels of functioning [21,22]. Thus, many patients might have difficulties accurately measuring these reductions and their levels of premorbid functioning in a self-report assessment. In addition to the DSQ, diagnosing a patient with ME/CFS should be conducted with an additional medical and psychological examination. Furthermore, the samples came from diverse case ascertainment methods as well as from different countries, and while this limits internal validity, it does improve external validity.

Our study illustrated an efficient way to measure substantial reductions in functioning in patients with ME/CFS. Often patients are the most reliable in determining their reductions in pre- and post-illness functionality, and the one item on the DSQ provides a simplified and valid method to capture this symptom.

## Figures and Tables

**Table 1:** Substantial reduction in ME/CFS versus control groups by method.

Method	ME/CFS % (N)	Control % (N)
1	91.2 (2,161)	13.6 (49)
2	89.0 (2,107)	4.7 (17)
3	92.2 (2,183)	10.9 (39)
4	90.8 (2,150)	7.5 (27)
5	91.7 (2,171)	6.4 (23)
